# The Influence of Contextual Variables on Training Load Prescription in Basketball: An Example of a Professional Australian Men’s Basketball Team

**DOI:** 10.5114/jhk/202812

**Published:** 2025-04-30

**Authors:** Jack Patterson, Russell Rayner, David L. Carey, Mathew O’Grady, Scott W. Talpey

**Affiliations:** 1 Sports Performance and Nutrition Research Group, La Trobe University, Melbourne, Victoria, Australia.; 2 School of Allied Health, Exercise and Sport Science, Charles Sturt University, Port Macquarie, New South Wales, Australia.; 3 Institute of Health and Wellbeing, Federation Universsity, Mt, Helen, Victoria, Australia

**Keywords:** coaching, sport science, training, practice, team sport

## Abstract

This study aimed to investigate the influence of contextual variables related to team performance, individual performance and scheduling on the external training load placed upon professional basketball players following a won compared to a lost game. Fifteen male professional basketball players from a single club competing in the Australia's top tier National Basketball League (NBL) during the 2023/2024 season participated in this study. Total player load, peak player load, player load per minute and the work to rest ratio derived from accelerometry were measures of external player load used in the analysis. Linear mixed models with the match outcome (win/loss), expected margin vs. outcome, days between games, and player efficiency as fixed effects, and player ID as a random intercept were employed. A statistically significant (p = 0.001) 62.46 au difference in total player load was observed following a win compared to a loss. However, when considering the random effects of an individual, individual performance, team performance and scheduling as fixed effects, a non-significant (p = 0.086) difference was observed with the individual player being the most influential variable. There were no statistically significant differences in peak player load (p = 0.734), player load per minute (p = 0.281), and the work to rest ratio (p = 0.782) following a win compared to a loss. The external training load prescribed to professional basketball players is highly individualized. Practitioners monitoring the training demands of players should consider the influence of individual factors when designing training.

## Introduction

Basketball is a physically demanding team-based invasion sport characterised by short bursts of high intensity activity such as sprinting, jumping, and changing directions followed by periods of recovery (Garcia et al., 2020; Pérez-Chao et al., 2023). Owing to the physically demanding nature of the sport, the prescription of an appropriate training load in basketball has a profound impact on the player’s performance, team performance, and injury risk (Arede et al., 2022; Weiss et al., 2017). Training load can be characterised as internal or external in nature. External training load represents the physical demands encountered by the player, whereas internal training load represents players’ physiological and psychological response to training, both of which provide valuable insight for practitioners when planning and monitoring training demands (Espasa-Labradoor et al., 2023; Scanlan et al., 2014; Svilar et al., 2018). For example, recent research by Coyne et al. (2021) highlighted that both internal and external training load variables collected on 13 International women’s basketball players in the leadup to the 2016 Olympic Games significantly correlated with coach ratings of individual performance during competition. Additionally, Weiss et al. (2017) reported that acute:chronic workload ratios between 1.00 and 1.49 were optimal for physical preparation and reduced injury risk in professional men’s basketball. For a thorough review on the relationship between training load and basketball performance the reader is directed to the systematic reviews by Petway et al. (2020) and Espasa-Labradoor et al. (2023). Nevertheless, a clear finding within the literature is that the dose of training placed upon a player has distinct implications for their performance.

However, a training load prescription can be influenced by a range of factors both individual and contextual. Individual factors are specific to the player (e.g. injury history, training age etc.), while contextual factors are those that are not part of the physical training process (e.g. team performance, scheduling etc.) (Nijland et al., 2024). Within the research, several studies have investigated the influence of contextual factors on the training load prescription. Contextual factors investigated within the literature range from the playing position (Dalton-Barron et al., 2021), the location of competition (Guerro-Calderon et al., 2021), to scheduling (Rago et al., 2023). Curtis et al. (2021) investigated the influence of the player’s status (starter vs. non-starter) on the accumulated training load in a large cohort of 107 National Collegiate Athletic Association (NCAA) male soccer players and found that starting players had a significantly greater training load placed upon them than non-starting players. In a similar study Curtis et al. (2020) reported that the outcome of a previous match and the day between matches significantly influenced the training load prescribed for the same population of collegiate soccer players. Specifically in basketball, competition demands have been shown to be significantly impacted by contextual factors such as the game outcome. Fox et al. (2019) reported that the number of jumps, high intensity accelerations, decelerations and changes of direction were significantly greater in the lost compared to the won game.

Research that has investigated the effects of contextual factors on training demands in basketball is scarce. However, a study conducted over the course of a season in a semi-professional Spanish men’s basketball team reported that players’ experience, the player’s position, the phase of the season and the upcoming opponent were contextual factors that significantly influenced the subjective internal training load using ratings of perceived exertion (Sansone et al., 2021). Similar results which also used a subjective rating of perceived exertion to determine internal training load have been reported in semi-professional female basketball players (Pinar et al., 2022). To date, no study in basketball has investigated the influence of contextual factors on external training load in professional players using wearable technology such as accelerometers and local positioning systems (LPSs). Local Positioning Systems provide information on a player’s movement using radio-frequency signals that measure the distance between a wearable device with an embedded accelerometer placed upon the athlete and anchor nodes distributed around the environment in which training or competition occurs (Luteberget et al., 2018). The use of LPS and accelerometry technology specifically for indoor sports such as basketball allows for robust valid and reliable external training load data that can be used to inform the safe and effective prescription of training. The purpose of this investigation was to determine whether external training load determined by accelerometery would be different following a won compared to a lost game when considering contextual variables related to the expected vs. actual margin of victory, individual player’s performance, and scheduling in Australian professional men’s basketball players. These contextual variables were selected for analysis due to their relationship with team and individual performance.

## Methods

### Participants

A convenience sample of fifteen male professional basketball players from a single club competing in the Australia's top tier National Basketball League (NBL) during the 2023/2024 season participated in this study. The mean age, body height and body mass for players were 26.7 ± 4.1 yrs, 1.98 ± 0.08 m and 97.8 ± 11.1 kg, respectively. A player's data were excluded if they were determined by the club's sports medicine team to be on a reduced training load for the session due to injury or illness.

### Design and Procedures

**Figure 1:**
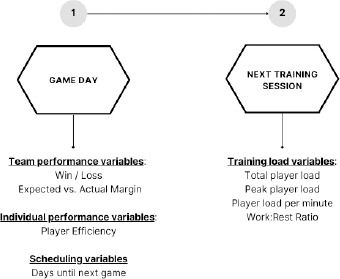
Schematic representation of the study design.

A retrospective study design was used to determine whether the external load in the training session immediately following competition in a professional men’s basketball team was significantly different following a won compared to a lost game. Player load data were collected from fifteen players competing in the Australia’s top tier professional basketball league for 19 training sessions (that immediately followed a competition) across the competition phase of the 2023/2024 season. The regular season in the Australia’s National Basketball League (NBL) consists of 28 games played from October to March. Within the regular season a team will typically play two games per week. The training session immediately following the game was chosen for analysis due to its proximity to the previous competition that can influence the training prescription. Even though training sessions following a competition are generally less fatiguing, they still include elements of technical skill refinement, tactical development and physical preparation contributing to an overall training load for the player. Figure 1 visually displays the design of the study, contextual variables and external training load variables for this investigation. This investigation received approval from the Human Research Ethics Committee of the La Trobe University, Melbourne, Victoria Australia (protocol code: HEC24314; approval date: 20 August 2024).

### Measures

Contextual variables related to team performance were the outcome of the previous game (win/loss), the expected vs. actual margin. The team performance variable of ‘expected vs. actual margin’ was determined by the difference in the projected outcome of the game based upon the market betting line at close of odds from the Bet365 website (www.bet365.com.au) and the actual outcome. For example, if the team was projected to win by a margin of 10 points (+10) and the team lost by a margin of five points (−5) the difference score would be −15 points. This contextual variable was chosen for inclusion in the analysis because of the nuanced nature of victory and defeat, highlighting the concept of a “bad win” or a “good loss”. Decisions regarding the prescription of subsequent training may be influenced by how well the team performed with respect to the anticipated outcome. Individual performance was determined by the player efficiency rating provided by the league website (www.nbl.com) as calculated by subtracting the negative contributions of the player during the game (missed field goals, missed free throws, and turnovers) from their positive contributions (points, field goals made, free throws made, assists, rebounds, blocks and steals) normalised per minute played during the game. ‘Days until the next game’ was a contextual variable related to scheduling and determined by calculating the number of days from the previous until the next game.

External training load variables were collected via a local positioning system (LPS) Catapult ClearsSky (Catapult, Melbourne, Australia) within the team's training facility. The LPS uses ground-based sensors to track the movement of players paired with a wearable Catapult Vector T7 unit with an embedded accelerometer (Catapult, Melbourne, Australia) worn by players in a custom-built harness firmly positioned between their shoulder blades at approximately the C7-T1 level. The wearable units have an inbuilt accelerometer, a gyroscope, and a magnetometer each sampling at 100 Hz. The variable of player load as the key external load metric for this investigation was determined by instantaneous data collected by the accelerometer, located within the unit worn by the player. The accelerations and decelerations across all axes (forward-backward, up-down, and side to side) over the time of training were summed to provide a robust indication of the individual “load” over the session using the following equation:


$$Player\;Load=\frac{\left[\sqrt{{\left(fwd_{t=i+1}-fwd_{t=1}\right)}^{2}}+\sqrt{{\left(side_{t=i+1}-side_{t=1}\right)}^{2}}+\sqrt{{\left(up_{t=i+1}-up_{t=1}\right)}^{2}}\right]}{100}$$

Derivatives of player load used for analysis in this investigation were peak player load, player load per minute and the work to rest ratio. Peak player load was determined as the rolling average of the player load over the duration of the training session with the most intense one-minute phase recorded. Player load per minute was determined as the absolute training load value for the session divided by the total number of minutes in the session. The work to rest ratio was determined by the time in the work phase divided by the time in the rest phase, where the work phase included periods of high intensity movement such sprinting, jumping and changing directions, while the rest phase included standing, walking and low- intensity jogging. Player load data using this system has been previously shown to demonstrate strong reliability and validity (Luteberget et al., 2018).

### Statistical Analysis

Linear mixed models with the match outcome (win/loss), the expected margin vs. outcome, days between games, and player’s efficiency as fixed effects, and the player’s ID as a random intercept were employed.

All analyses were conducted within the statistical computing and data visualisation program R Studio (Version 4.4.11; R Core Team, 2024) within the RStudio environment using the ‘readxl’, ‘dplyr’ and ‘ggplot2’, ‘lme4’ and ‘sjplot’ packages. Additionally, Cohen’s *d* effects sizes were calculated to determine the magnitude of differences in player load variables following a won compared to a lost game (Cohen, 2013).

## Results

The mean (± standard deviation) for external training load variables collected for the 19 training sessions and following a win or a loss is presented in Table 1.

**Table 1:** Mean ± standard deviation and Cohen’s *d* effect size for all external training load variables.

Variable	All training sessions	Following a win	Following a loss	Cohen’s *d*
Player load (au)	412.7 ± 140.2	450.0 ± 133.3*	388.2 ± 139.7	0.4 (small)
Peak player load (au)	4.9 ± 1.7	5.1 ± 1.6	4.8 ± 1.8	0.1 (small)
Player load / min (au)	6.7 ± 1.5	6.5 ± 1.5	6.9 ± 1.5	0.2 (small)
W:R ratio (au)	0.3 ± 0.1	0.3 ± 0.1	0.3 ± 0.1	0.0 (small)

W:R = work to rest ratio. * = significantly different from a loss (p < 0.05)

**Figure 2:**
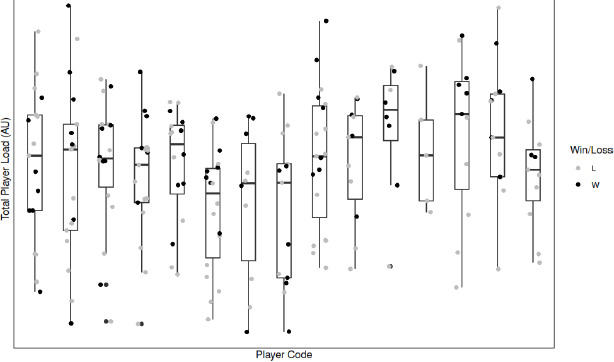
Total player load following a win compared to a loss for individual players. *L = Loss; W= Win*

**Figure 3:**
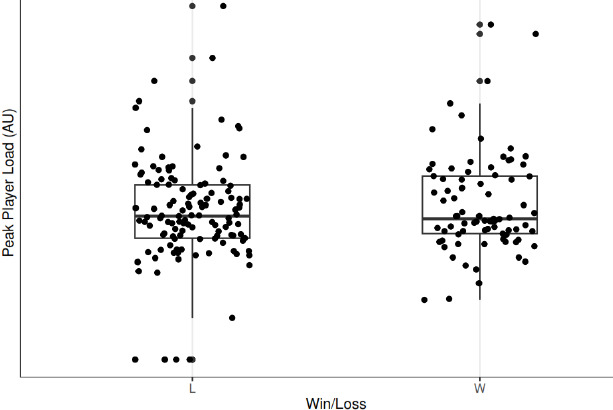
Peak player load following a win compared to a loss. *L = loss; W = Win*

**Figure 4:**
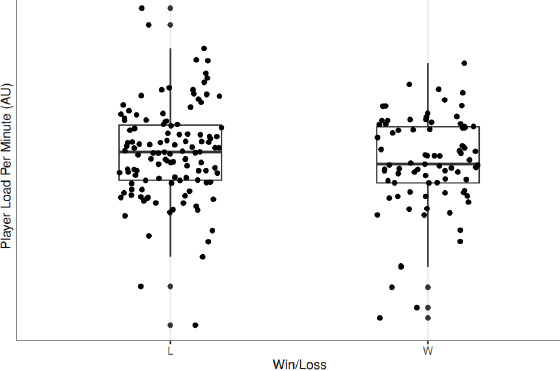
. Player load per minute following a win compared to a loss. *L = loss; W = Win*

**Figure 5:**
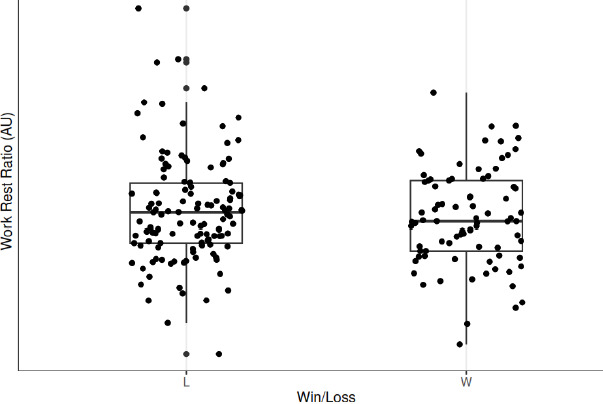
Work to rest ratio following a win compared to a loss. *L = Loss; W = Win*

Without consideration for the influence of the individual player, the margin vs. expected outcome, and time to the next game, there was a statistically significant (*p* = 0.001) 62.46 au difference in total player load following a win compared to following a loss. However, when contextual variables were included as a random effect, there was a non-significant (*p* = 0.086) difference in total player load following a win compared to a loss. The contextual variable with the greatest influence on total player load was individual player. Additionally, there were no significant differences in peak player load (*p* = 0.734), player load per minute (*p* = 0.289), and the work to rest ratio (*p* = 0.782). These changes in player load variables following a win vs. following a loss are visually represented in Figures 2–5.

## Discussion

The purpose of this investigation was to quantify the differences in the external load placed upon professional basketball players in training following a won compared to a lost game. There is limited previous research on the effect of contextual factors on the training load prescription in basketball, and the few studies that have been conducted in this area focused on semi-professional athletes. A key finding from this investigation is that although there was a statistically significant difference in the total player load in the subsequent training session following a won compared to a lost game, this difference was negated when contextual factors were included as random effects into the analysis. Therefore, it can be stated that in this case, the load placed upon players on this team was not affected by contextual variables influencing the training prescription. Interestingly, the most influential factor on the external load prescribed in the training session was the individual player themselves.

The finding that the external training load prescription was highly individualised is in alignment with fundamental principles of training. The principle of individualisation refers to planning and implementation of training that aligns with the specific needs of the individual (Pickering and Kiely, 2019). Previous research quantifying training and game demands of National Basketball Association (NBA) players across the season also supports the individualised nature of the training prescription in elite basketball (Russell et al., 2021). Russell and colleagues (2021) reported that the external training load prescribed to professional basketball players was strongly influenced by the players’ status as starters or non-starters, with starting players having significantly greater weekly load. Additionally, players having 3–5 years of experience were prescribed significantly greater weekly loads than those with 6–9 and 10+ years of experience. However, the study by Russell et al. (2021) evaluated the training load placed upon players across different types of sessions such as those focused on the development of technical and tactical components as well as recovery and fitness training. Additionally, it is important to note that three games per week are common practice in congested scheduling within the NBA, while in the NBL teams typically play two games per week. The training sessions analysed within the current investigation were a blend of recovery, technical and tactical development.

The individual player’s performance as determined by the league’s player efficiency statistic was not an influential factor in the external load in training following a won compared to a lost game. This finding is somewhat surprising, but can be rationalised through the measure of individual performance employed in the study. Since the current study utilised a retrospective analysis of data, the player efficiency statistic was the sole measure of individual performance used. Previous research by Coyne et al. (2021) used coaches’ ratings as a holistic approach to evaluate basketball player’s performance because it considered the players’ role within the tactical game model of the team and reported that the internal and external loads in training strongly correlated with the coaches subjective rating of the players’ performance. Additionally, previous research that evaluated individual player performance in team sport using subjective coaches’ ratings reported that coach perceptions of players’ performance were significantly influenced by the external load metrics of game time and the percentage of game time spent running at a high speed (Bauer et al., 2015). This finding that players’ efficiency in competition was not an influential contextual factor in the external load of players in training following a competition provides impetus for future research to explore the use of coaches’ ratings as a more holistic approach to evaluating basketball player performance.

The lack of statistically significant differences in players’ load metrics following a win compared to a loss may also be linked to the phase of training in which the data were collected from. The phase of a periodised training program is an influential factor in the load placed upon a player. Previous research has shown that training loads are greatest during general preparation phases of a program as coaches attempt to elicit an increase in players’ fitness (Aoki et al., 2017). The data analysed for the current investigation were from the competition phase of the team’s periodised training program. A previous study in professional basketball has shown that the external load placed upon players during the competition phase is significantly lower than in the preparation phase, and that there is less fluctuation in load between particular training sessions (Aoki et al., 2017). The consistency in the training load prescription during the competition phase may be linked to an increased emphasis on technical and tactical components of performance and maintenance of physical fitness resulting in greater cognitive than physical demands (Champion et al., 2023).

There were some limitations to the current investigation that need to be discussed alongside the results. Firstly, accelerometer-based player load metrics were the only external load measures used for analysis in this investigation. Due to varying training environments for the team, consistent availability of LPS derived measures of additional external load metrics such as the amount and intensities of accelerations, decelerations, changes of directions and jumps was not possible. Previous research in elite soccer has indicated that player load is strongly correlated with the total distance covered by a player in training and competition (Oliva-Lozano et al., 2022) and therefore, the incorporation of additional external load metrics may provide the sport scientist with further insight into how team, individual and scheduling factors influence external training load. Secondly, robust monitoring of the load placed upon an athlete should consider all types of loads to ascertain the stress placed upon an athlete (Gabbett, 2016). Due to the retrospective nature of the current investigation, there were no measures of internal or cognitive load for the training sessions that immediately followed a competition. Since the training sessions analysed in this investigation were a blend of recovery, technical and tactical development, it is possible that following the competition the coach of the team may have designed training sessions that aimed to improve team performance through activities that possessed high cognitive fidelity focusing on decision making demands similar to competition resulting in high cognitive demands rather than physical demands (Champion et al., 2023). Future research should aim to include the cognitive load demands of basketball training sessions alongside internal and external measures of physical load when evaluating training demands. Lastly, this investigation was conducted on a single team of male professional Australian basketball players and these findings are specific to this team. However, these results provide insight into the training demands of elite basketball players, which is lacking within the peer-reviewed literature.

## Conclusions

The purpose of this investigation was to explore the influence of contextual factors related to team performance, individual performance and scheduling on the external load prescribed in training following competition. The results highlight that the most influential factor on the prescription of external load is the individual themselves. From a practical application standpoint, when prescribing external training loads practitioners should be cognizant of the individual player considerations and aim to plan activities that align with the individual needs from a physical, technical and tactical perspective. Sport scientists should work collaboratively with the head coach in these instances to ensure physical training demands integrate with the coach’s objectives for the training session.

## References

[ref1] Aoki, M. S., Ronda, L. T., Marcelino, P. R., Drago, G., Carling, C., Bradley, P. S., & Moreira, A. (2017). Monitoring training loads in professional basketball players engaged in a periodized training program. Journal of Strength & Conditioning Research, 31(2), 348–358. 27243913 10.1519/JSC.0000000000001507

[ref2] Arede, J., Freitas, T. T., Johnson, D., Fernandes, J. F., Williams, S., Moran, J., & Leite, N. (2022). Training load, maturity timing, and future national team selection in national youth basketball players. Journal of Functional Morphology and Kinesiology, 7(1), 21. 35225907 10.3390/jfmk7010021 PMC8883974

[ref3] Bauer, A. M., Young, W., Fahrner, B., & Harvey, J. (2015). GPS variables most related to match performance in an elite Australian football team. International Journal of Performance Analysis in Sport, 15(1), 187–202.

[ref4] Champion, L., Middleton, K., & MacMahon, C. (2023). Many Pieces to the Puzzle: A New Holistic Workload Approach to Designing Practice in Sports. *Sports Medicine-Open*, 9(1), 38. 37256515 10.1186/s40798-023-00575-7 PMC10232382

[ref5] Cohen, J. (2013). Statistical power analysis for the behavioral sciences. Routledge.

[ref6] Coyne, J. O., Coutts, A. J., Newton, R. U., & Haff, G. G. (2021). Relationships between different internal and external training load variables and elite international women’s basketball performance. International Journal of Sports Physiology and Performance, 16(6), 871–880. 33631715 10.1123/ijspp.2020-0495

[ref7] Curtis, R. M., Huggins, R. A., Benjamin, C. L., Sekiguchi, Y., Adams, W. M., Arent, S. M., ... & Casa, D. J. (2020). Contextual factors influencing external and internal training loads in collegiate men's soccer. Journal of Strength & Conditioning Research, 34(2), 374–381. 31524781 10.1519/JSC.0000000000003361

[ref8] Curtis, R. M., Huggins, R. A., Benjamin, C. L., Sekiguchi, Y., Arent, S. M., Armwald, B. C., ... & Casa, D. J. (2021). Seasonal accumulated workloads in collegiate men's soccer: A comparison of starters and reserves. Journal of Strength & Conditioning Research, 35(11), 3184–3189. 31453937 10.1519/JSC.0000000000003257

[ref9] Dalton-Barron, N. E., McLaren, S. J., Black, C. J., Gray, M., Jones, B., & Roe, G. (2021). Identifying contextual influences on training load: An example in professional Rugby Union. Journal of Strength & Conditioning Research, 35(2), 503–511. 29979279 10.1519/JSC.0000000000002706

[ref10] Espasa-Labradoor, J., Calleja-González, J., Montalvo, A. M., & Fort-Vanmeerhaeghe, A. (2023). External Load Monitoring in Female Basketball: A Systematic Review. Journal of Human Kinetics, 88, 173–198. 10.5114/jhk/166881 PMC10407319 37559766

[ref11] Fox, J. L., Stanton, R., Sargent, C., O’Grady, C. J., & Scanlan, A. T. (2019). The impact of contextual factors on game demands in starting semiprofessional male basketball players. International Journal of Sports Physiology and Performance, 15(4), 450–456. 31605525 10.1123/ijspp.2019-0203

[ref12] Gabbett, T. J. (2016). The training—injury prevention paradox: should athletes be training smarter and harder?. British Journal of Sports Medicine, 50(5), 273–280. 26758673 10.1136/bjsports-2015-095788 PMC4789704

[ref13] García, F., Vázquez-Guerrero, J., Castellano, J., Casals, M., & Schelling, X. (2020). Differences in physical demands between game quarters and playing positions on professional basketball players during official competition. Journal of Sports Science & Medicine, 19(2), 256. 32390718 PMC7196749

[ref14] Guerrero-Calderón, B., Klemp, M., Castillo-Rodriguez, A., Morcillo, J. A., & Memmert, D. (2021). A new approach for training-load quantification in elite-level soccer: contextual factors. International Journal of Sports Medicine, 42(08), 716–723. 33321524 10.1055/a-1289-9059

[ref15] Luteberget, L. S., Spencer, M., & Gilgien, M. (2018). Validity of the Catapult ClearSky T6 local positioning system for team sports specific drills in indoor conditions. *Frontiers in Physiology*, 9, 115. 29670530 10.3389/fphys.2018.00115 PMC5893723

[ref16] Nijland, R., Toering, T., Watson, C. G., de Jong, J., & Lemmink, K. A. (2024). A Scoping Review on the Influence of Contextual Factors on Training Load in Adolescent Soccer Players: What Do We Know? *Sports*, 12(7), 172. 39058063 10.3390/sports12070172 PMC11280791

[ref17] Oliva-Lozano, J. M., Conte, D., Fortes, V., & Muyor, J. M. (2023). Exploring the use of player load in elite soccer players. Sports Health, 15(1), 61–66. 35034515 10.1177/19417381211065768 PMC9808829

[ref18] Pérez-Chao, E. A., Portes, R., Gómez, M. Á., Parmar, N., Lorenzo, A., & Jiménez Sáiz, S. L. (2023). A Narrative Review of the Most Demanding Scenarios in Basketball: Current Trends and Future Directions. Journal of Human Kinetics, 89, 231–245. 38053946 10.5114/jhk/170838 PMC10694712

[ref19] Petway, A. J., Freitas, T. T., Calleja-González, J., Medina Leal, D., & Alcaraz, P. E. (2020). Training load and match-play demands in basketball based on competition level: A systematic review. *PloS One*, 15(3), e0229212. 32134965 10.1371/journal.pone.0229212 PMC7058381

[ref20] Pickering, C., & Kiely, J. (2019). The development of a personalised training framework: Implementation of emerging technologies for performance. Journal of Functional Morphology and Kinesiology, 4(2), 25. 33467340 10.3390/jfmk4020025 PMC7739422

[ref21] Piñar, M. I., García, D., Mancha-Triguero, D., & Ibáñez, S. J. (2022). Effect of situational and individual factors on training load and game performance in Liga Femenina 2 Basketball female players. *Applied Sciences*, 12(15), 7752.

[ref22] Rago, V., Mohr, M., & Vigh-Larsen, J. F. (2023). Quantifying training load and intensity in elite male ice hockey according to game-related contextual variables. Biology of Sport, 40(1), 283–289. 36636188 10.5114/biolsport.2023.114282 PMC9806747

[ref23] Russell, J. L., McLean, B. D., Stolp, S., Strack, D., & Coutts, A. J. (2021). Quantifying training and game demands of a National Basketball Association season. *Frontiers in Psychology*, 12, 7932. 10.3389/fpsyg.2021.793216 PMC8724530 34992569

[ref24] Sansone, P., Ceravolo, A., & Tessitore, A. (2021). External internal perceived training loads and their relationships in youth basketball players across different positions. International Journal of Sports Physiology and Performance, 17(2), 249–255. 34583325 10.1123/ijspp.2020-0962

[ref25] Scanlan, A. T., Wen, N., Tucker, P. S., & Dalbo, V. J. (2014). The relationships between internal and external training load models during basketball training. Journal of Strength & Conditioning Research, 28(9), 2397–2405. 24662233 10.1519/JSC.0000000000000458

[ref26] Svilar, L., Castellano, J., & Jukić, I. (2018). Load monitoring system in top-level basketball team: Relationship between external and internal training load. Kinesiology, 50(1), 25–33.

[ref27] Weiss, K. J., Allen, S. V., McGuigan, M. R., & Whatman, C. S. (2017). The relationship between training load and injury in men’s professional basketball. International Journal of Sports Physiology and Performance, 12(9), 1238–1242. 28253031 10.1123/ijspp.2016-0726

